# Finding ATF4/p75NTR/IL-8 Signal Pathway in Endothelial–Mesenchymal Transition by Safrole Oxide

**DOI:** 10.1371/journal.pone.0099378

**Published:** 2014-06-06

**Authors:** Di Ge, Qingchuan Jing, Wenbo Zhao, Hongwei Yue, Le Su, ShangLi Zhang, Jing Zhao

**Affiliations:** 1 Shandong Provincial Key Laboratory of Animal Cells and Developmental Biology, School of Life Science, Shandong University, Jinan, China; 2 Institute of Poultry Science, Shandong Academy of Agricultural Sciences, Jinan, China; VCU, United States of America

## Abstract

Targeting the endothelial-to-mesenchymal transition (EndoMT) may be a novel therapeutic strategy for cancer and various diseases induced by fibrosis. We aimed to identify a small chemical molecule as an inducer of EndoMT and find a new signal pathway by using the inducer. Safrole oxide (SFO), 50 µg/ml, could most effectively induce EndoMT within 12 h. To understand the underlying molecular mechanism, we performed microarray, quantitative real-time PCR and western blot analysis to find key factors involved in SFO-induced EndoMT and demonstrated the involvement of the factors by RNAi. The expression of activating transcription factor 4 (ATF4), p75 neurotrophin receptor (p75NTR), and interleukin 8 (IL-8) was greatly increased in SFO-induced EndoMT. Knockdown of ATF4 inhibited the SFO-induced EndoMT completely, and knockdown of p75NTR or IL-8 partially inhibited the EndoMT, which suggests that all three factors were involved in the process. Furthermore, knockdown of p75NTR inhibited the SFO-increased IL-8 expression and secretion, and knockdown of ATF4 inhibited SFO-increased p75NTR level significantly. The ATF4/p75NTR/IL-8 signal pathway may have an important role in EndoMT induced by SFO. Our findings support potential novel targets for the therapeutics of cancer and fibrosis disease.

## Introduction

The endothelial-to-mesenchymal transition (EndoMT) has been known as a critical process in heart development, such as in cardiac cushion morphogenesis [1]. EndoMT-derived cells are now known to function as fibroblasts in damaged tissue and therefore have an important role in tissue remodelling and fibrosis [2], [3]. Furthermore, in tumours, EndoMT is an important source of cancer-associated fibroblasts, which are known to facilitate tumour progression [4]. Recently, chemical small molecules that control differentiation in stem cells have been identified and are useful for investigating the mechanisms of cell fate decision [5]. Using some chemical small molecules to regulate EndoMT could help clarify the specific mechanisms of EndoMT, which might provide a therapeutic strategy for cancer and various other diseases associated with EndoMT [6].

In our laboratory, we synthesized safrole oxide (SFO), which has piperonyl and epoxy structures that are important in many compounds with physiological activity. We previously observed that 5 to 25 µg/ml SFO inhibited but 50 to 100 µg/ml promoted apoptosis of human umbilical vein endothelial cells (HUVECs) [7], [8]. Furthermore, at low concentrations, SFO could induce HUVEC transdifferentiation into neuron-like cells when it suppressed cell apoptosis in the absence of serum and fibroblast growth factor (FGF) [9]. Therefore, SFO might be an important small molecule affecting HUVEC apoptosis and transdifferentiation depending on its concentration. Strikingly, with high concentrations of SFO, HUVECs had an appearance of elongated-like mesenchymal cells. However, whether SFO can induce EndoMT at high concentrations is not clear.

In this study, we examined the function of SFO in EndoMT and explored the key factors involved in SFO-induced EndoMT. Especially, we lack reports about the function of activating transcription factor 4 (ATF4), p75 neurotrophin receptor (p75NTR) and interleukin 8 (IL-8) regulated by SFO in EndoMT. These three proteins were all involved in endothelial cell apoptosis [10]–[12]. During embryological development and throughout life, apoptosis often appeared accompanied with transdifferentiation, suggesting a strong association between apoptosis and transdifferentiation [13]. We elucidated the roles and the relationship of these three proteins in SFO-induced EndoMT.

## Materials and Methods

### Reagents

Medium M199 (31100-035) and fetal bovine serum (FBS, 10437036) were obtained from Gibco (USA). 3,4-(methylenedioxy)-1-(2′,3′-epoxypropyl)-benzene, or safrole oxide (SFO), was synthesized by the reaction of safrole with 3-chloroperoxybenzoic acid and purified by silica gel column chromatography [14]. It was dissolved in ethanol and applied to cells so that the final concentration of ethanol in the culture medium was <0.01% (vol/vol). Ethanol at 0.1% (vol/vol) did not affect cell viability [7], [14]. Antibodies for ATF4, p75NTR, alpha-smooth muscle actin (α-SMA), CD31, endothelial nitric oxide synthase (eNOS), GAPDH, β-actin and horseradish peroxidase-conjugated secondary antibodies as well as ATF4, p75NTR, and IL-8 siRNA were all from Santa Cruz Biotechnology (Santa Cruz, CA). IL-8 and C-X-C ligand 1 (CXCL1) ELISA kits were from R&D (USA).

### Cell culture and treatment

Investigations conformed to the principles outlined in the Declaration of Helsinki, and all protocols were approved by the Shandong University ethics review board. Primary human umbilical vein endothelial cells (HUVECs) were isolated from the human umbilical vein as described [15]. All experiments were performed on the cells from 10 to 20 passages. The MS1 cell line (a mouse pancreatic islet endothelial cell line) was obtained from the American Type Culture Collection (Manassas, VA) and grown in DMEM. Vascular smooth muscle cells (VSMCs) were obtained and cultured in M199 as described [16]. Cells were divided into 2 groups when the cultures of cells reached sub-confluence: controls, cultured in normal medium with 10% serum and 5 ng/ml FGF 2 (FGF-2); and SFO-treated, treated with normal medium (10% serum, 5 ng/ml FGF-2) and different concentrations of SFO for 3 to 12 h. SFO was dissolved in ethanol as previously reported [8].

### Morphometric analysis

SFO-treated HUVECs were washed with M199, and morphological changes were observed under a phase contrast microscope (Nikon, Japan) and quantified (100 cells per sample) by use of ImageJ (http://rsbweb.nih.gov/ij/plugins/circularity.html). Cell circularity, whereby a circular cell equals 0 and increasing elongation approaches 1, was calculated as follows:
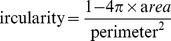



### Quantitative Real-time PCR (qPCR)

Cells were treated with 50 µg/ml SFO for the indicated times. Total RNA was extracted from cells with use of TriZol Reagent (Invitrogen Life Technologies). RNA (250–500 ng) was reverse-transcribed by use of M-MLV reverse transcriptase (Promega, USA). Forward (F) and reverse (R) primers designed for human genes were as follows: a-SMA-F 5′-TCATCCTCCCTTGAGAAGAG-3′, a-SMA-R 5′-ATGCCAGGGTACATAGTGGT-3′; CD31-F 5′-CTGAGGGTGAAGGGGGACAGGAC-3′, CD31-R 5′-AGTATTTTGCTTCTGGGGAC-3′; eNOS-F 5′-CATGTTTGTCTGCGGCGATG-3′, eNOS-R 5′-AAAGCTCTGGGTGCGTATGC-3′; IL-8-F 5′-AGGGTTGCCAGATGCAATAC-3′, IL-8-R 5′-GTGGATCCTGGCTAGCAGAC-3′; ATF4-F 5′-CATTCCTCGATTC CAGCAAAGCAC-3′, ATF4-R 5′-TTCTCCAACATCCAATCTGTCCCG-3′; p75NTR-F 5′-CAACCCTGCAAAGGACTGTT-3′, p75NTR-R5′-CACCCAGACTCTGTCCCACT-3′. qPCR reactions involved use of the QuantiTect SYBR Green PCR kit (QIAgen) and LightCycler 2.0 (Roche Diagnostics). Reactions were carried out in a 25 µl volume containing 12.5 µl of 2×SYBR Green PCR Master Mix. The fold-changes for RNA level were calculated by use of MxPro 4.00 (Stratagene).

### Western blot analysis

Western blot analysis was as described [17]. Briefly, total protein was extracted by use of protein lysis buffer (Beyotime Institute of Biotechnology, China). The protein concentration was quantified by bicinchonininc acid protein assay (Beyotime Institute of Biotechnology). An amount of 40 µg protein was run on 12% SDS-polyacrylamide gel. Then proteins in gels were transferred to nitrocellulose membranes, which were blocked in 5% (w/v) nonfat dry milk or bovine serum albumin (for phosphorylated protein only) in phosphate buffered saline (PBS) with Tween 20 (PBST; 0.05%) for 1 h, then incubated with primary antibodies (1∶1000) at 4°C overnight. Membranes were washed in PBST and incubated with horseradish peroxidase-conjugated secondary antibodies (1∶5000) at room temperature for 1 h. Immunoreactive protein bands were developed by use of an enhanced chemiluminescence kit (Thermo Electron Corp., Rockford, IL, USA). GAPDH or β-actin was a loading control.

### Cell contraction analysis

The medium for HUVECs treated with 50 µg/ml of SFO for 6 h was replaced with basal M199 medium (without FGF and FBS) after one wash with the same medium. Then HUVECs were treated with sphingosylphosphorylcholine (SPC, 30 µM), an inducer of VSMC contraction [16]. The contractile changes of cells at 1 h were quantified by use of Leica software; VSMCs were a positive control.

### Microarray Analysis

Total RNA was extracted from cells treated with 50 µg/ml SFO for 6 h with use of TRIzol reagent (Invitrogen, USA). RNA samples underwent whole-human–genome oligo microarray assay by CapitalBio Co., (Beijing; http://www.capitalbio.com). The detailed experimental procedures for microarray and data analyses were previously reported [18]. We considered genes differentially expressed with >1.5-fold change in the ratio of expression between SFO-treated and ethanol control cells. The microarray results represent a single experiment. The data were deposited at Gene Expression Omnibus (Accession no. GSE55149).

### Knockdown of ATF4, p75NTR and IL-8

Knockdown of ATF4, p75NTR and IL-8 was performed as described [19]. An amount of 40 nM ATF4 and IL-8 and 60 nM p75NTR siRNA was transfected into HUVECs by use of RNAiFect Transfection Reagent (Qiagen, Hilden, Germany). The same dose of scramble siRNA was used as a control. Silencing efficiency was verified by qPCR and western blot analysis [20].

### Statistical analyses

All data are expressed as means ± SE from at least 3 independent experiments. Differences between multiple groups were analyzed by one-way ANOVA. Statistical significance was considered at p<0.05. Data analysis involved use of SPSS 12.0 (SPSS Inc., Chicago, IL).

## Results

### SFO promoted morphological change of HUVEC

**Figure 1:**
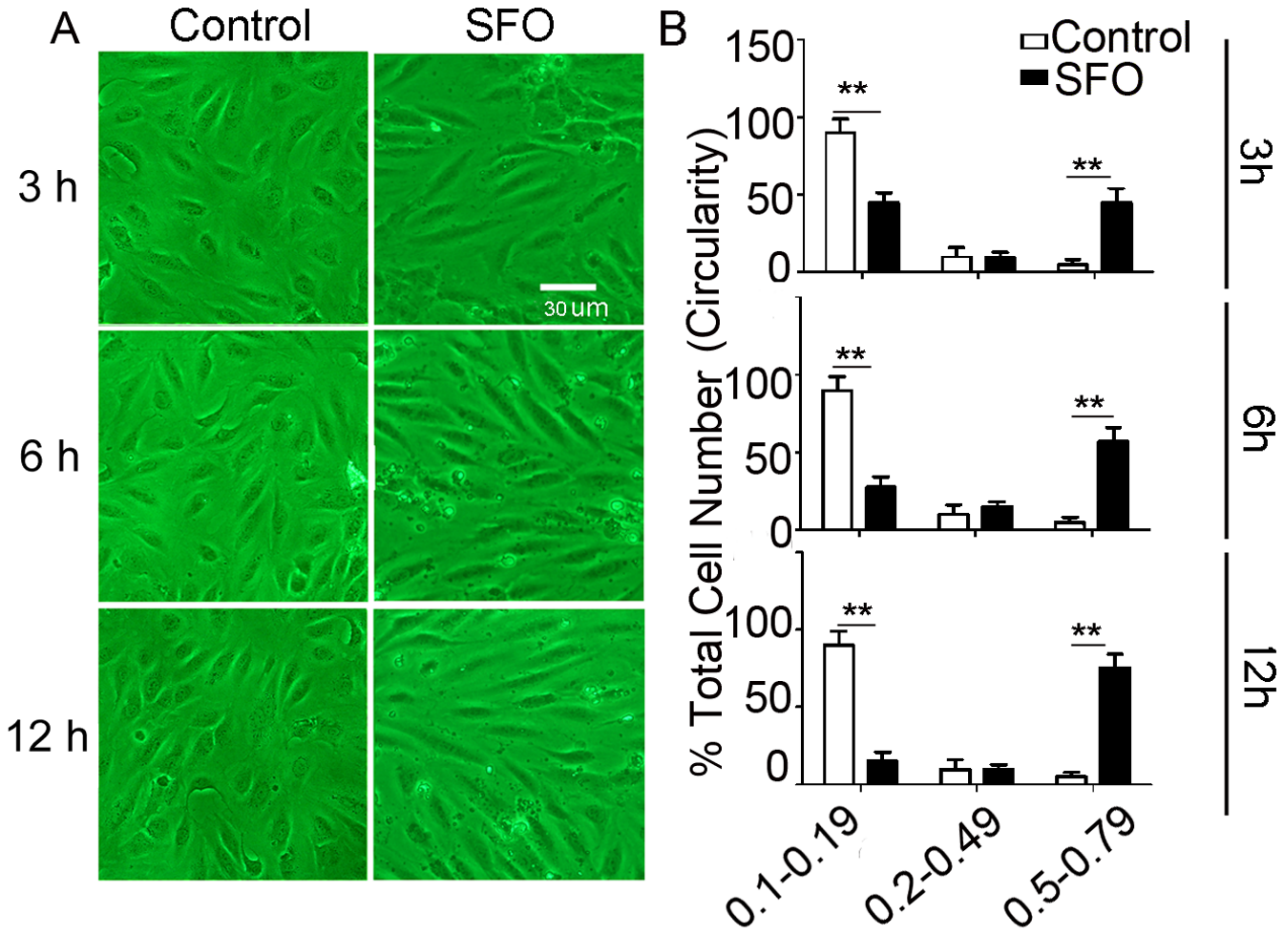
Phase-contrast photomicrographs (A) and morphometric analyses (B) of human umbilical vascular endothelial cells (HUVECs). HUVECs were cultured in normal medium without or with 50 µg/ml safrole oxide (SFO) for 3, 6 and 12 h. (A) Cells in the control group retained cobblestone features, whereas cells cultured with SFO had elongated features and were arranged into “streamlined” structures. (B) Cell circularity, where a circle equals 0 and increasing elongation approaches 1, was calculated after SFO treatment (**p<0.01, n = 5).

Control HUVECs showed a cobblestone morphology. When HUVECs were exposed to a high concentration of SFO (50–70 µg/ml) for 3, 6 or 12 h, cells increasingly showed an elongated appearance. Furthermore, the cells were aligned, to create a “streamlined” effect with extended treatment [21] (**Fig. 1A**). Circularity analysis showed that HUVECs stimulated by 50 µg/ml SFO were more elongated, with circularity measurement ranging from 0.2 to 0.7 (p<0.01) (**Fig. 1B**). The phenomenon was sustained for 12 h. After then, smooth muscle-like cells undergo apoptosis [8].

### SFO promoted EndoMT

**Figure 2:**
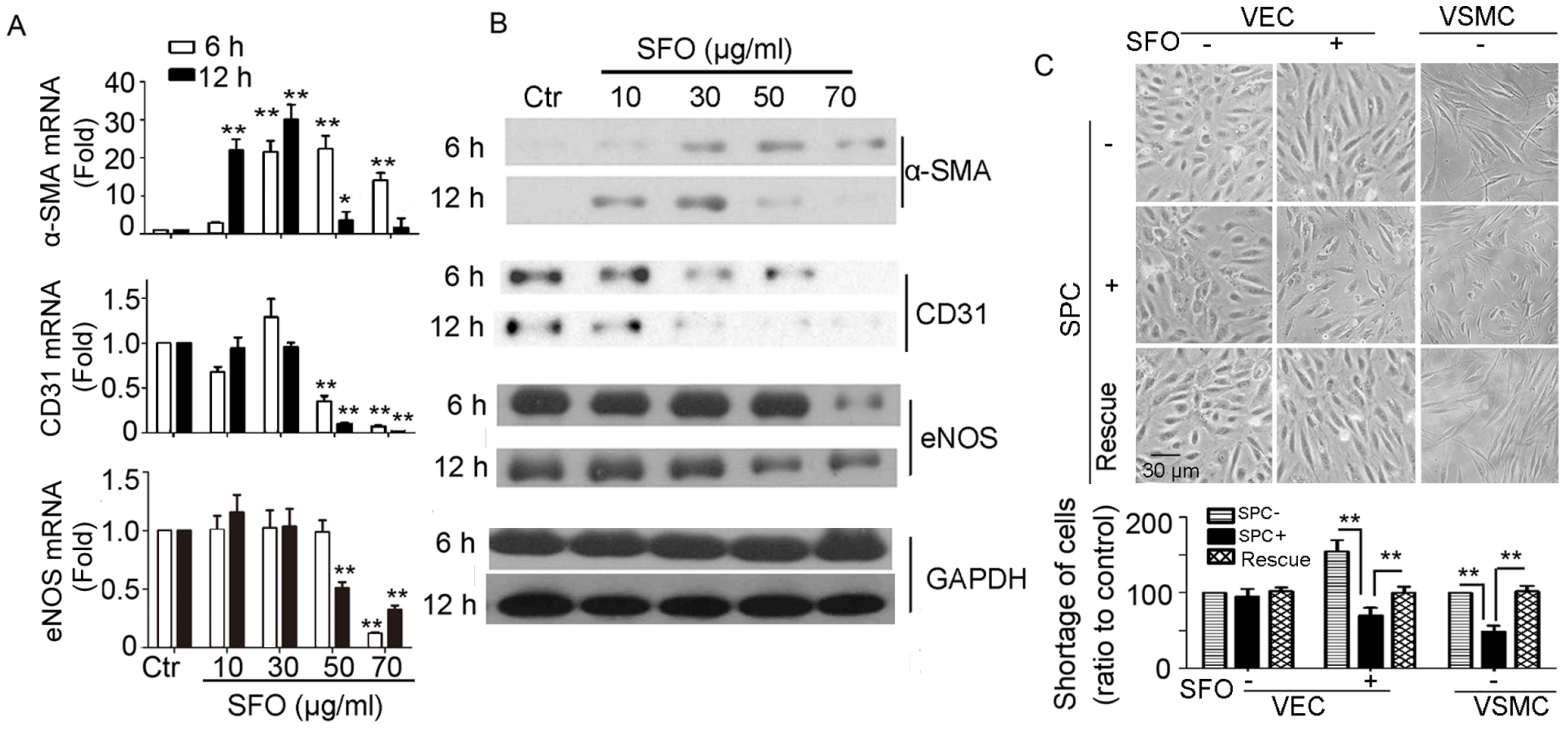
SFO promoted endothelial–mesenchymal transition (EndoMT) in HUVECs. HUVECs were exposed to 10–70 µg/ml SFO in normal medium. (A) qPCR analysis of mRNA expression of α-SMA, CD31 and eNOS in HUVECs treated with SFO at 3, 6 and 12 h. Expression was normalized to that of GAPDH. *p<0.05;**p<0.01 vs. Ctr, n = 4. (B) Western blot analysis of protein expression of α-SMA, CD31 and eNOS in HUVECs treated with SFO at 6 and 12 h. Results are representative of 3 independent experiments. (C) HUVECs were treated with ethanol (Control) or 50 µg/ml of SFO for 6 h, then exposed to 30 µM sphingosylphosphorylcholine (SPC) for 1 h, with or without rescue for 1 h, and contraction of cells was calculated. VSMC stimulated with SPC was as a positive control (**p<0.01, n = 3).

To identify SFO as an inducer of EndoMT in the presence of serum and FGF-2, we examined the specific markers of mesenchymal and endothelial cells in HUVECs treated with SFO at different concentrations. α-SMA, the specific marker of smooth muscle cells and EndoMT [22], [23], showed a very low level of expression in untreated cells. In contrast, low concentrations of SFO (10–30 µg/ml) treatment significantly increased α-SMA expression at 6 and 12 h (**Fig. 2A and B**). High concentrations of SFO (50–70 µg/ml) increased α-SMA expression only before 12 h. At 12 h, it could not increase α-SMA expression again, and this time point was verified to induce apoptosis in our previous report [7], [8]. In contrast, levels of the specific marker of endothelial cells, CD31, and eNOS were decreased by high but not low concentrations of SFO (**Fig. 2A and 2B**). Along with the morphological changes and our previous findings [7], [8], we concluded that high concentrations of SFO promoted EndoMT within 12 h.

SPC is an inducer of VSMC contraction [16]. To further understand whether these EndoMT cells have contraction ability, we stimulated cells with SPC, 30 µM, for 1 h and found that α-SMA-expressing cells induced by SFO contracted as compared with real VSMCs. When deprived of SPC, both SFO-induced EndoMT cells and real VSMCs recovered. In contrast, HUVECs without SFO treatment showed no response to SPC (**Fig. 2C**). These functional detection data verified that SFO induced transdifferentiation of HUVECs to VSMCs.

### ATF4, p75NTR and IL-8 levels were elevated during SFO-induced EndoMT

**Figure 3:**
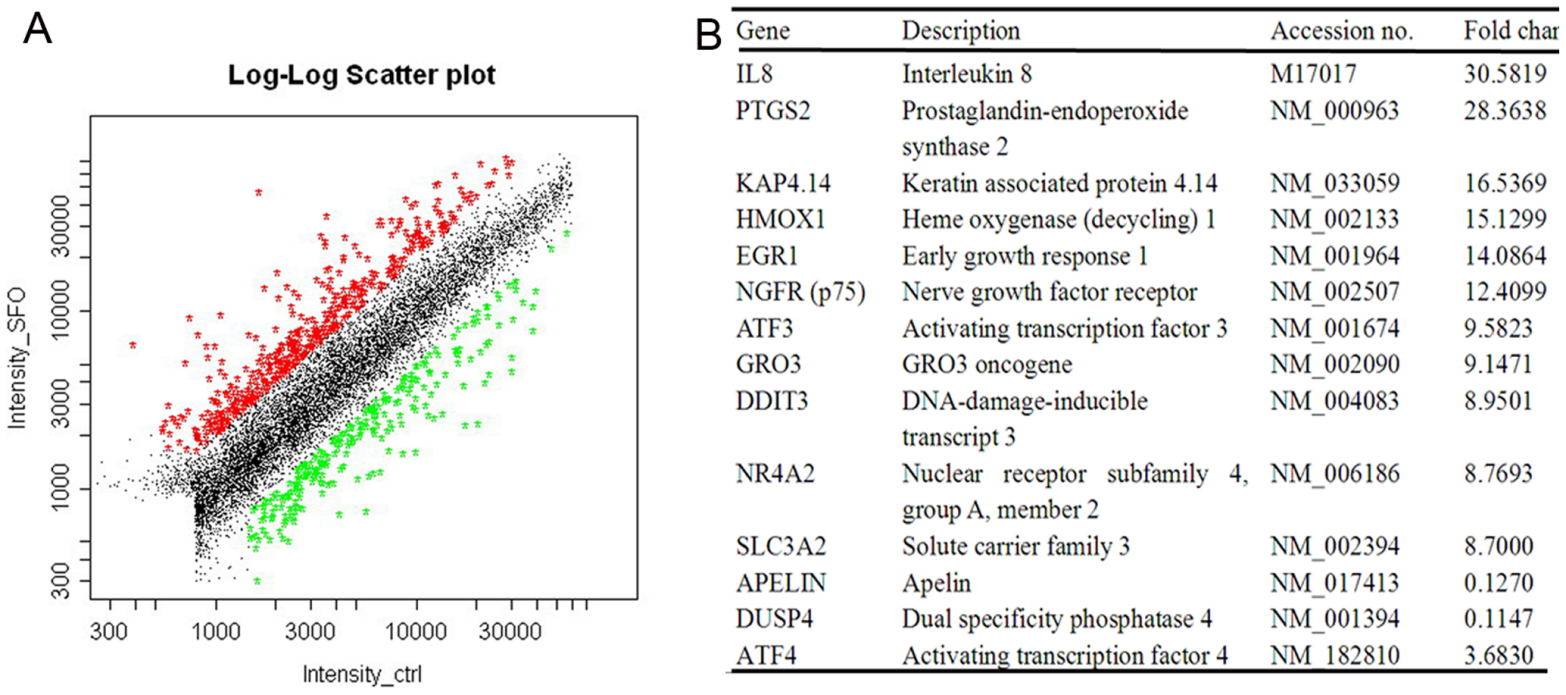
Microarray assay of gene expression induced by 50 µg/ml of SFO in medium for 6 h. (A) Scatter plot of differentially expressed genes. (B) The 13 genes with more than eight-fold change in expression and ATF4 on microarray analysis. The microarray results represent a single experiment.

**Figure 4:**
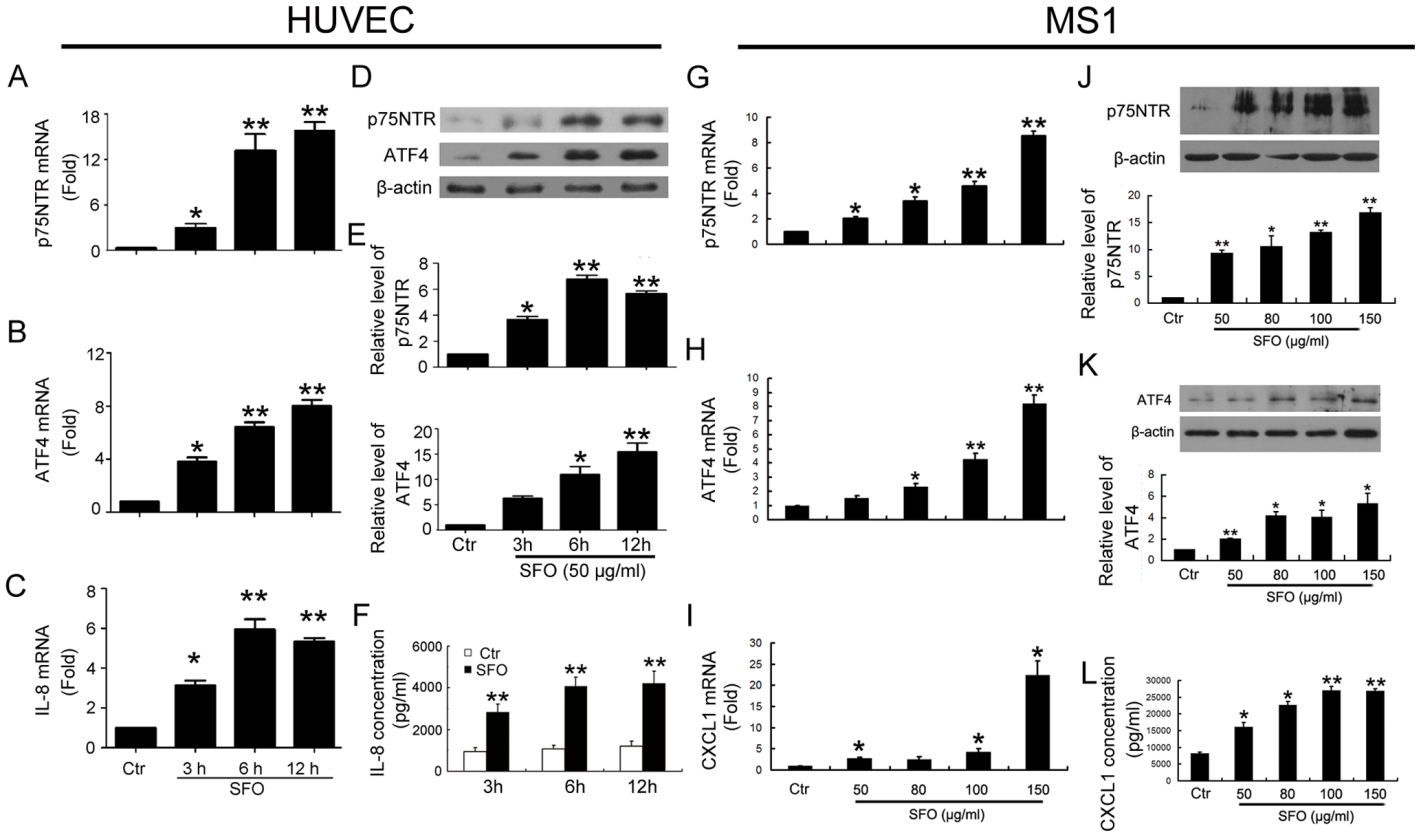
ATF4, p75NTR and IL-8 were increased by SFO in HUVECs and MS1 cells. HUVECs were exposed to 50 µg/ml of SFO at 3, 6 and 12 h, then the relative mRNA level of the three genes was detected by qPCR (A to C) and western blot analysis (D and E) or ELISA (F). Data below the western blot bands were used to quantify the density of the bands. β-actin was a normalization control. MS1 cells were exposed to 50–150 µg/ml SFO at 6 h (The effective time and concentration used in this study was according to our preliminary experiment), the relative mRNA level and protein level of ATF4, p75NTR and IL-8 were detected by qPCR (G to I) and western blot analysis (J and K). IL-8 secretion was detected by ELISA (L). β-actin was used as a normalization control (*p<0.05; **p<0.01 vs. Ctr, n = 3).

To understand the molecular mechanism by which SFO induces EndoMT, we first found the candidates that were implicated in the EndoMT. On microarray assay, 717 genes showed changed expression (>1.5-fold) during EndoMT induced by treatment with SFO, 50 µg/ml, for 6 h; 13 genes showed > eight-fold changed expression (**Fig. 3**). The greatly increased expression of ATF4, p75NTR and IL-8 (CXCL1 in mouse) was verified by qPCR and western blot assay in HUVECs and endothelial MS1 cells (**Fig. 4**).

### Effect of ATF4, p75NTR and IL-8 knockdown on α-SMA expression in HUVECs induced by SFO

**Figure 5:**
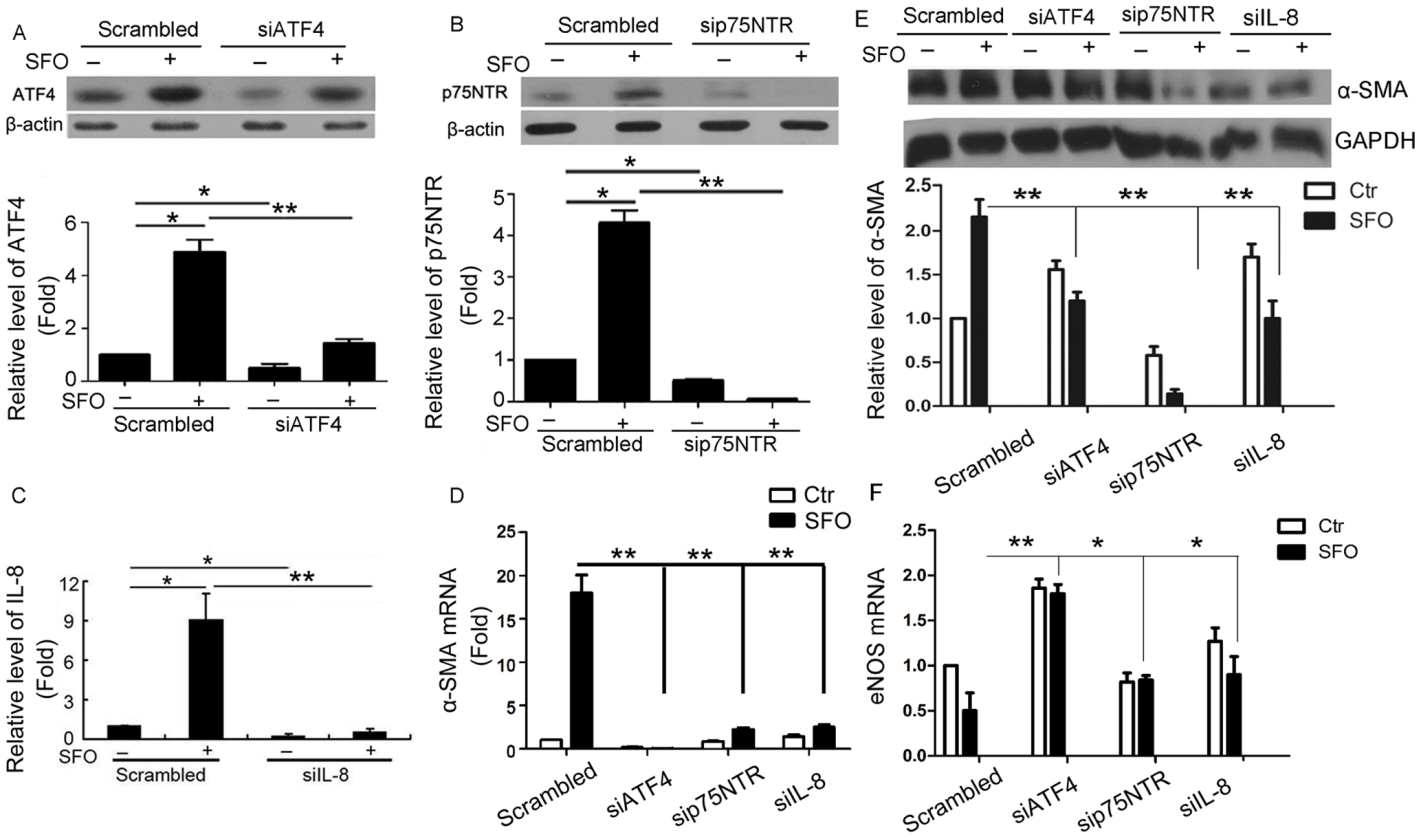
ATF4, p75NTR and IL-8 participated in SFO-induced EndoMT in HUVECs. Cells were preincubated with 40 nM ATF4, IL-8 or 60 nM p75NTR siRNA or scramble siRNA for 48 h, then 50 µg/ml SFO for 3 h for qPCR and 6 h for western blot assay. (A and B) Western blot analysis of siRNA-mediated downregulation of ATF4 and p75NTR in HUVECs. Data below the western blot bands were used to quantify the density of the bands. (C) qPCR analysis of siRNA-mediated downregulation of IL-8. GAPDH was a normalization control. (D and E) Analysis of effect of ATF4, p75NTR and IL-8 siRNA on SFO-induced α-SMA in HUVECs by qPCR (D) and western blot (E). (F) qPCR analysis of siRNA effects on eNOS expression in HUVECs. Expression was normalized to that of GAPDH. (*p<0.05, **p<0.01, n = 3).

To further confirm the role of ATF4, p75NTR and IL-8 in SFO-increased α-SMA expression, we transfected ATF4, p75NTR and IL-8 siRNA into HUVECs followed by treatment with SFO. Transfection of the selected siRNAs significantly inhibited the expression of the corresponding genes (**Fig. 5A to C**). Knockdown of ATF4, p75NTR and IL-8 significantly inhibited SFO-induced α-SMA in mRNA and protein levels (**Fig. 5D and E**). Furthermore, knockdown of ATF4, p75NTR and IL-8 rescued SFO-inhibited eNOS expression (**Fig.5F**).

### ATF4 siRNA inhibited p75NTR expression and p75NTR siRNA inhibited IL-8 expression in HUVECs

**Figure 6:**
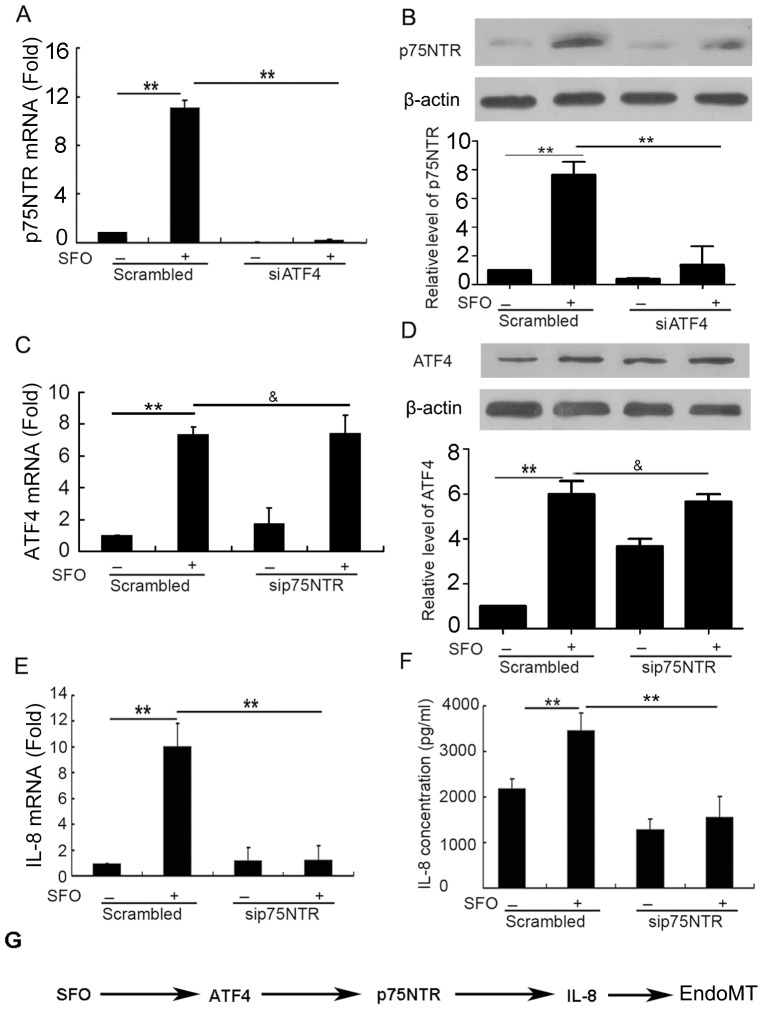
The ATF4/p75NTR/IL-8 signaling pathway was involved in the induction of EndoMT by SFO. (A and B) Cells were preincubated with ATF4 siRNA for 48 h, then 50 µg/ml SFO for 3 h for qPCR assay and 6 h for western blot assay. Effect of ATF4 siRNA on SFO-induced p75NTR expression in HUVECs was detected by qPCR assay (A) and western blot assay (B). Data below gels were used to quantify the density of the bands. (C to F) Cells were preincubated with p75NTR siRNA for 48 h, then 50 µg/ml of SFO for 3 h for qPCR assay and 6 h for western blot or ELISA assay. Effect of p75NTR siRNA on SFO-induced ATF4 expression in HUVECs was detected by qPCR assay (C) and western blot assay (D). Effect of p75NTR siRNA on SFO-induced IL-8 expression and release in HUVECs was detected by qPCR assay (E) and ELISA (F). G. A schematic of the signaling pathways of SFO in HUVEC transdifferentiation. (**p<0.01; & p>0.05, n = 3).

Transcription factors play an important role in the control of genes. Therefore, we detected whether ATF4 could regulate the expression of p75NTR. Suppressing the role of ATF4 inhibited SFO-increased p75NTR mRNA and protein levels (**Fig. 6A, B**), which suggested that ATF4 is upstream of p75NTR in SFO-induced EndoMT in HUVECs.

We analyzed the effect of p75NTR on ATF4 and found that p75NTR siRNA did not affect its level in SFO-treated HUVECs. So we verified that p75NTR is downstream of ATF4 in HUVECs treated with SFO during EndoMT (**Fig. 6C and D**). Furthermore, p75NTR knockdown decreased the mRNA level of IL-8 in SFO-treated HUVECs at 3 h and secretion at 6 h (**Fig. 6E and F**). The results suggested that p75NTR acts upstream of IL-8 in SFO-induced EndoMT in HUVECs.

## Discussion

EndoMT participates in the development and vascular or microvascular damage in many kinds of diseases, such as pathogenesis of atherosclerosis and the progression of renal fibrosis [2], [3]. Here we first identified SFO as an inducer of EndoMT in the presence of serum and FGF-2 to mimic the *in vivo* condition, to determine whether SFO could provide a useful chemical tool to study the molecular mechanisms of the EndoMT for therapeutics for the treatment of cancer and fibrosis disease. VEGF blockage by SU5416 was reported to cause endothelial cells differentiation into neuronal-like cells and smooth muscle-like cells at the same time [24]. Our data agreed with the report that low concentration of SFO induced VEC differentiation to neuron-like cells, but a high concentration induced EndoMT transition. Whether neural cells and smooth muscle cells originate from the same pluripotent cells still needs further research.

Next, we performed a microarray assay to determine candidates that may be involved in EndoMT induced by SFO. Among the genes with changed expression, ATF4 is a master regulator for evolutionarily conserved mammalian stress response pathways [25]. ATF4 has a pro-apoptotic role in endothelial cells by activating a signalling cascade involving CHOP and ATF3 [12]. However, its role on transdifferentiation is not clear. In our study, knockdown of ATF4 inhibited SFO-induced EndoMT completely, which suggests that ATF4 is involved in EndoMT. Furthermore, ATF4 was reported to regulate the expression of IL-8 [26]. In this study, we further found that ATF4 can regulate the expression of p75NTR, and p75NTR can regulate the expression of IL-8, which might be the new mechanism for ATF4 participating in EndoMT (**Fig. 6G**).

IL-8, a member of the CXC chemokine family, modulating permeability, migration, proliferation and apoptosis of endothelial cells [11], [27]. In this study, we found that IL-8 was also involved in EndoMT. IL-8 is a potent chemoattractant for neutrophils and, also triggers firm adhesion of rolling monocytes to the vascular endothelium, thus participating in the pathogenesis of many inflammatory diseases [28]. Our data suggest that IL-8 might regulate these diseases by inducing EndoMT. In the current study, all changes in IL-8 expression were detected in the medium but not during cell lysis. Furthermore, IL-8 levels in the medium were increased by SFO when transdifferentiation was promoted and decreased with p75NTR siRNA treatment when transdifferentiation was hampered. So IL-8 might affect transdifferentiation by a paracrine effect. Further study is needed to determine whether HUVECs treated with IL-8 and/or SFO in the presence of neutralizing IL-8 antibodies can verfity this paracrine effect.

Among the genes with changed expression, p75NTR protein is unique located at the cell membrane. Neurotrophin low-affinity p75NTR induces apoptosis of endothelial cells and VSMCs and impairs angiogenesis [10]. However, p75NTR is a marker for regenerating fibers in inflamed and dystrophic muscle, which has a broad transcriptional repertoire associated with muscle development and maturation [29]. In this study, knockdown of p75NTR could partially inhibit the EndoMT, which suggests that p75NTR is an important regulator of the EndoMT.

Previously, we found that a high concentration of SFO was a powerful inducer of HUVEC apoptosis [7], [8]. In this study, we found that SFO induced EndoMT transition before apoptosis in HUVECs in the presence of serum and FGF-2. *In vivo*, terminally differentiated cells develop increased sensitivity to apoptogens [30]. For example, in thioacetamide-induced rat liver injury and subsequent fibrosis, well-differentiated myofibroblasts expressing α-SMA may disappear by apoptosis for healing, whereas myofibroblasts without α-SMA expression do not die [31]. Furthermore, retinoids and all-trans-retinoic acid were able to induce differentiation and late apoptosis in pancreatic cancer cells and acinar tissue *in vitro*, respectively [32]. Thus, the phenomenon of transdifferentiation before apoptosis might be an important mechanism for apoptosis of high-differentiation potency cells.

In summary, our experiments showed that SFO could induce EndoMT in the presence of serum and FGF-2, for a useful tool for understanding the mechanism of EndoMT. ATF4, p75NTR and IL-8 all participated in the process of EndoMT induced by SFO, and they modulated the EndoMT by an ATF4/p75NTR/IL-8 pathway. These results may reveal a molecular mechanism related to HUVEC transdifferentiation. Furthermore, our data suggest that transdifferentiation appearing before apoptosis might be meaningful for cell apoptosis by providing increased sensitivity to apoptogens.
